# Alternative Glycerol Balance Strategies among *Saccharomyces* Species in Response to Winemaking Stress

**DOI:** 10.3389/fmicb.2016.00435

**Published:** 2016-03-31

**Authors:** Roberto Pérez-Torrado, Bruno M. Oliveira, Jana Zemančíková, Hana Sychrová, Amparo Querol

**Affiliations:** ^1^Food Biotechnology Department, Systems Biology in Yeast of Biotechnological Interest, Instituto de Agroquímica y Tecnología de los Alimentos, IATA-CSICValencia, Spain; ^2^Department of Membrane Transport, Institute of PhysiologyCAS, Prague, Czech Republic

**Keywords:** glycerol, yeast, *Saccharomyces*, stress, winemaking

## Abstract

Production and balance of glycerol is essential for the survival of yeast cells in certain stressful conditions as hyperosmotic or cold shock that occur during industrial processes as winemaking. These stress responses are well-known *in S. cerevisiae*, however, little is known in other phylogenetically close related *Saccharomyces* species associated with natural or fermentation environments such as *S. uvarum, S. paradoxus* or *S. kudriavzevii*. In this work we have investigated the expression of four genes (*GPD1, GPD2, STL1*, and *FPS1*) crucial in the glycerol pool balance in the four species with a biotechnological potential (*S. cerevisiae; S. paradoxus; S. uvarum;* and *S. kudriavzevii*), and the ability of strains to grow under osmotic and cold stresses. The results show different pattern and level of expression among the different species, especially for *STL1*. We also studied the function of Stl1 glycerol symporter in the survival to osmotic changes and cell growth capacity in winemaking environments. These experiments also revealed a different functionality of the glycerol transporters among the different species studied. All these data point to different strategies to handle glycerol accumulation in response to winemaking stresses as hyperosmotic or cold-hyperosmotic stress in the different species, with variable emphasis in the production, influx, or efflux of glycerol.

## Introduction

In the fermentation industry, especially winemaking, the resistance to osmotic stress and the ability to grow at low temperatures are required features for yeast strains (Pretorius et al., 2012). It is known that *S. cerevisiae* seeks to increase intracellular glycerol content when subjected to osmotic stress or cold in vinification or standard laboratory growth conditions (Panadero et al., 2006; Petelenz-Kurdziel et al., 2013; Oliveira et al., 2014). This intracellular accumulation is very important for osmotic equilibrium during the first phase of fermentation and to act as key cryoprotector agent for adaptation to cold environments allowing cellular viability with implications in the fermentation yield (Remize et al., 2001; Tulha et al., 2010). A rapid and specific activation of the gene expression have been identified as an essential mechanism in the *S. cerevisiae* cells to respond to acute stresses, such as those associated with the different industrial fermentation processes (de Nadal et al., 2011). However, little is known about these stress responses in other *Saccharomyces* species associated with natural or fermentation environments such as *S. uvarum* (Naumov et al., 2002; Rementeria et al., 2003; Demuyter et al., 2004), *S. paradoxus*, isolated from Croatian vineyards (Redzepovic et al., 2002) or natural yeast hybrids between species of the genus *Saccharomyces* such as *S. cerevisiae* × *S. kudriavzevii* (Gonzalez et al., 2007) and *S. cerevisiae* × *S. uvarum* (Le Jeune et al., 2007; Pérez-Torrado et al., 2015) which may participate in the fermentative processes. *S. uvarum* and *S. kudriavzevii* present important physiological traits like the ability to grow at lower temperatures and produce more glycerol than *S. cerevisiae* (Gonzalez et al., 2007; Gamero et al., 2013; Oliveira et al., 2014). However, *S. paradoxus*, besides being a widely distributed yeast species mainly associated with natural environments and not very relevant in fermentations, is physiologically more similar to *S. cerevisiae* (Tronchoni et al., 2009).

It is well-known that *S. cerevisiae* and other yeast species are capable to modulate the glycerol synthesis and its intracellular content in accordance with environmental osmotic changes (Hohmann et al., 2007; Hubmann et al., 2011). They can also control an active glycerol import from the extracellular medium in symport with protons via Stl1 transporter (Tulha et al., 2010; Dušková et al., 2015a). Besides its important role in osmoregulation, the Stl1 function is directly related to cell survival and adaptation to cold stress in *S. cerevisiae* strains (Tulha et al., 2010). The yeast cells may also regulate their glycerol content by controlling its efflux via the Fps1 channel (Luyten et al., 1995). This channel can be quickly closed avoiding the glycerol efflux, and thus contributing to an efficient osmoregulation with direct implications on increasing the fermentation yield (Wei et al., 2013).

The understanding of the phylogenetic and physiological relationships between *S. cerevisiae* and other *Saccharomyces* species, as well as main ecological, environmental, and human factors that have driven the emergence of phenotypic changes among species of *Saccharomyces* genus, have been cleared in many works (Landry et al., 2006; Peris et al., 2014). Several studies have focused in understanding the cryophilic character of *S. uvarum* and specially *S. kudriavzevii* at the molecular level, including transcriptomic and metabolomic studies (Combina et al., 2012; López-Malo et al., 2013). Some aspects of *S. kudriavzevii* have been highlighted in relation to cold resistance and winemaking as membrane composition (Tronchoni et al., 2012), or translation efficiency (Tronchoni et al., 2014). However, little information about these species and the glycerol synthesis is available. In the case of *S. kudriavzevii*, the increased cold tolerance has been related to elevated glycerol synthesis as a consequence of increased expression and activity of Gpd1p in winemaking conditions (Oliveira et al., 2014). For this reason a better understanding of *Saccharomyces* species physiological and molecular features with potential biotechnological interest is needed.

Hence, in this work we decided to investigate the expression of genes crucial to the balance of glycerol (*GPD1, GPD2, STL1*, and *FPS1*) in two yeast strains of each of the four species with a biotechnological potential (*S. cerevisiae*; *S. paradoxus*; *S. uvarum;* and *S. kudriavzevii*). We also studied the function of Stl1 glycerol symporter, in the survival to osmotic changes and cell growth capacity in winemaking environments.

## Methods

### Yeast strains and growth conditions

Yeast strains origin, availability are described in Table 1. Two different strains of each species were studied. For *S. cerevisiae*, T73 model wine strain (Querol et al., 1994; Lopes et al., 2010) and the commercial wine strain Fermol Cryophile FCry (AEB Group); selected as adapted to low temperature (Gamero et al., 2013) were chosen. The 108 and Chr 16.2 strains isolated from natural environment were used as representatives of *S. paradoxus*. For *S. uvarum*, the 12600 and BMV58 strains isolated from wine in Spain were studied. BMV58 was commercialized (Lallemand Inc) because of its high glycerol production and good fermentative properties (patent ES2330709 B1). For *S. kudriavzevii* species, IFO1802 (type strain), and the CR85 wild strain isolated in Spain (Dušková et al., 2015b) were used. The *S. cerevisiae* BY4741Δ*hog*1Δ*stl1* (Pérez-Torrado et al., 2009) was used as a laboratory strain for the expression of *STL1* genes and comparison of the function of their products under hyperosmotic-stress conditions.

**Table 1:** **Strains used in this study**.

**Strain**	**Species**	**Description**
T73^a^	*S. cerevisiae*	Wine strain, Alicante, Spain
FCry	*S. cerevisiae*	Wine strain, commercial (AEB), France
Chr 16.2	*S. paradoxus*	Wild strain, Oak bark, Hungary
108	*S. paradoxus*	Wild strain, Croatia
BMV58	*S. uvarum*	Wine, Spain
12600^a^	*S. uvarum*	Sweet wine, Spain
CR85	*S. kudriavzevii*	Wild strain, Oak bark, Spain
IFO1802^b^	*S. kudriavzevii*	Type strain, Soil, Japan
BY4741*hog1Δstl1Δ*	*S. cerevisiae*	Lab strain (Dušková et al., 2015b)
BY-hs-YEp352	*S. cerevisiae*	BY4741*hog1Δstl1Δ*YEp352 (This work)
BY-hs-p*STL1*-T73	*S. cerevisiae*	BY4741*hog1Δstl1Δ*YEp352-*STL1*_T73_(This work)
BY-hs-p*STL1*-BMV58	*S. cerevisiae*	BY4741*hog1Δstl1Δ*YEp352-*STL1*_BMV58_(This work)
BY-hs-p*STL1*-IFO1802	*S. cerevisiae*	BY4741*hog1Δstl1Δ*YEp352-*STL1*_IFO1802_(This work)

*Some strains are available from collections*.

a CECT;

b *NBRC*.

Yeast cells were maintained and grown in YPD medium (2% glucose, 2% Bacto peptone, and 1% Yeast extract) or SC-Ura medium (YNB 0.67%, glucose 2%, Drop-out –Ura 1.92 g/l (Formedium)) at 28°C for the *S. cerevisiae* and *S. paradoxus* species and 25°C for *S. kudriavzevii* and *S. uvarum* species.

The wine fermentations were performed in 250 ml bottles filled with 200 ml of MS300 synthetic must (100 g/L glucose, 100 g/L fructose, 6 g/L citric acid, 6 g/L malic acid, mineral salts, vitamins, anaerobic growth factors, 300 mg/L assimilable nitrogen) simulating standard grape juice (Bely et al., 2003) at 12°C with agitation (150 rpm) in triplicate. Overnight precultures were inoculated at 5.0 × 10^6^ cells/ml density determined by measuring OD_600_. To study the expression of genes related to glycerol balance under hyperosmotic stress, the cells from exponentially growing precultures were washed with water and transferred to YP (2% Bacto peptone and 1% yeast extract) with 2% glucose or 2% mannitol as a source of carbon, to the same medium supplemented with 1 M sorbitol (hyperosmotic stress), which is not assimilable for any of the species studied, or to H_2_O (hypoosmotic stress). This experiment was performed in 2 l flasks filled with 400 ml of media in triplicate at 25°C and 150 rpm.

The tolerance to hyperosmotic stress was evaluated by drop tests. Yeasts were grown overnight in YPD or SC-ura medium, then cultures were diluted to OD_600_ = 0.2 and cells were allowed to grow in the same media until OD_600_ = 1. Then, serial dilutions of cells were transferred to plates with YPD; YPD + 0.8 M NaCl; YPD + 1.25 M KCl, incubated at 12 and 25°C and evaluated each day. The growth of *Saccharomyces* species was also compared in plates with SC containing 2 M sorbitol or 2 M KCl and supplemented or not with 1 mM glycerol. To investigate the functional differences of Stl1, the growth of BY4741Δ*hog*1Δ*stl1* cells transformed with appropriate plasmids was monitored on plates with SC-ura containing 0.7 M sorbitol, which or 0.3 M KCl and with or without 10 mM glycerol. Experiments were performed in triplicate, representative results are shown.

For the intracellular glycerol measurements, cells were grown in 250 ml flasks with SC-ura with 10% of glucose at 28°C with agitation (150 rpm) in triplicate until the glucose concentration achieved < 2g/l.

### Plasmid construction

Plasmids expressing the *S. cerevisiae* T73, *S. bayanus* BMV58, and *S. kudriavzevii* IFO1802 *STL1* genes under *NHA1* gene promoter were constructed by exchanging the *NHA1* coding sequence in pNHA1-985 (YEp352 derivative, Kinclová et al., 2001) by homologous recombination. All constructions were confirmed by diagnostic PCR and sequencing. The primers, listed in Table 2, were designed based on data from *Saccharomyces* Genome Database (Cherry et al., 2012) and used to amplify the DNA fragments (from genomic DNAs) with suitable flanking regions for homologous recombination and confirmation.

**Table 2:** **Primers used in this study**.

**Name**	**Sequence**	**Purpose**	**Species**
GPD1-F	TGTGGTGCTTTGAAGAACG	qPCR and sequencing	*S.c., S.u., S.p., S.k*.
GPD1-R	GTTTCTTCTCTAGATTCTGG	qPCR and sequencing	*S.c., S.u., S.p., S.k*.
GPD2-F	GTTCCACAGACCWTACTTCC	qPCR	*S.c., S.u., S.p., S.k*.
GPD2-R	CCATCCCATACCTTCTACG	qPCR	*S.c., S.u., S.p., S.k*.
FPS1-F	GTTTTGYGTTTTTCCAAAGC	qPCR	*S.c., S.u., S.p., S.k*.
FPS1-R	TGATAAGCCATRGARGCATT	qPCR	*S.c., S.u., S.p., S.k*.
STL1-F	GCTTATTGGATTGATTTTGGG	qPCR	*S.c., S.u., S.p*.
STL1-R	TGTTAACAGCATCGTGAAGC	qPCR	*S.c., S.u., S.p*.
STL1-F	ACAGCATCGTGAAGCATAGC	qPCR	*S. kudriavzevii*
STL1-R	TGGCTGATTTCTCAAAGTCG	qPCR	*S. kudriavzevii*
ACT1-F	CATGTTCCCAGGTATTGCCG	qPCR	*S.c., S.u., S.p., S.k*.
ACT1-R	GCCAAAGCGGTGATTTCCT	qPCR	*S.c., S.u., S.p., S.k*.
18S-F	TTGCGATAACGAACGAGACC	qPCR	*S.c., S.u., S.p., S.k*.
18S-R	CATCGGCTTGAAACCGATAG	qPCR	*S.c., S.u., S.p., S.k*.
P-NHA1	CAACTCTGTGTGATATAG	Verification	*S. cerevisiae*
ScSTL1 - R2	CAACCCTGTTCCAACACC	Verification	*S. cerevisiae*
ScSTL1 - F2	GGACAGTCCGGTTGGGGTTG	Verification	*S. cerevisiae*
SbSTL1 - F2	CTACCCTGAAACTGCTGG	Verification	*S. uvarum*
SbSTL1 - R2	GCCCAGTAGTCACGGAAAGC	Verification	*S. uvarum*
SkSTL1 - F2	CCCTGAAACCGCTGGTAG	Verification	*S. kudriavzevii*
SkSTL1 - R2	GCCTTGGACATTTCGGAC	Verification	*S. kudriavzevii*
YEp352-R	GGGGATGTGCTGCAAGGCG	Verification	
YEp-SbSTL1-F	GTACATTATAAAAAAAAATCCTGAACTTAGCTAGATATTATGAAGGAATCAAAAGTATCTAAG	Cloning YEp352	*S. uvarum*
YEp-SbSTL1-R	CACGACGTTGTAAAACGACGGCCAGTGCCAAGCTTGCATGTTACTTTTCAGAGCTGTTTTCAT	Cloning YEp352	*S. uvarum*
YEp-ScSTL1-F	GTACATTATAAAAAAAAATCCTGAACTTAGCTAGATATTATGAAGGATTTAAAATTATCG	Cloning YEp352	*S. cerevisiae*
YEp-ScSTL1-R	CACGACGTTGTAAAACGACGGCCAGTGCCAAGCTTGCATGTCAACCCTCAAAATTTGCTT	Cloning YEp352	*S. cerevisiae*
YEp-SkSTL1-F	GTACATTATAAAAAAAAATCCTGAACTTAGCTAGATATTATGAGGAAATCAAAAGTATC	Cloning YEp352	*S. kudriavzevii*
YEp-SkSTL1-R	CACGACGTTGTAAAACGACGGCCAGTGCCAAGCTTGCATGCTAGTTTTCGGAATTTGGTTTC	Cloning YEp352	*S. kudriavzevii*

### Analytical determinations

The extracellular glycerol concentrations and residual sugars (glucose and fructose) were determined in must and medium samples by HPLC (Thermo Fisher Scientific, Waltham, MA) equipped with a refraction index detector. The column employed was a HyperREZTM XP Carbohydrate H+ 8 μm (Thermo Fisher Scientific) and the conditions used in the analysis were as follows: eluent, 1.5 mM H_2_SO_4_; flux, 0.6 ml/min; and oven temperature, 50°C. The samples were diluted, filtered through a 0.22-μm nylon filter (Symta, Madrid, Spain) and injected in duplicate.

To determine intracellular glycerol content, 5 OD_600_ units were harvested by filtration and quickly washed with 5 ml of water and transferred to a tube containing 1 ml of cold water. After no more than 20 s after sampling, the yeast suspension was boiled for 10 min, cooled on ice, and centrifuged at 15,300 × g for 10 min (4°C). The supernatant was collected, filtered and directly analyzed by HPLC. A second sample (5 OD_600_ units) was harvested by filtration in cellulose membrane, 25 mm pore size 0.45 μm (MF-Milipore) previously dried in the microwave at 350W for 20 min. and weighed. To determine dry weight, the cells in the membrane were carefully washed with 1 ml of water and dried under the same conditions. The values obtained are expressed as μg of glycerol per mg of yeast cells. Experiments were performed in triplicate.

### Gene expression determination

For each culture, 10–20 ml sample was taken at different times. The cells were quickly harvested by centrifugation, washed and frozen in liquid N_2._Then, frozen cells were lysed and homogenized by FastPrep-24 (MP Biomedicals) in LETS buffer (10 mm Tris pH 7.4, 10 mM lithium-EDTA, 100 mM lithium chloride, 1% lithium lauryl sulfate) with acid-washed glass beads (0.4 mm diameter; Sigma-Aldrich) for 30 s six times alternating with ice incubation. Total RNA was extracted and purified using the phenol:chloroform method with minor modifications (Combina et al., 2012). Then the RNA was converted to cDNA and the expression of *GPD1, GPD2, STL1*, and *FPS1* genes was quantified by qRT-PCR (quantitative real-time PCR). The cDNA strand was constructed using 2 μg of RNA mixed with 0.8 mM dNTP's, 80 pmol Oligo (dT) in 13 μl. The mixture was heated to 65°C for 5 min and quenched on ice for 1 min. 5 mM dithiothreitol (DTT), 50 U of RNase inhibitor (Invitrogen), 1 × First Strand Buffer (Invitrogen) and 200 U Superscript III (Invitrogen) were added to the 20 μl mixture, which was incubated at 50°C for 60 min and the reaction was inactivated after 15 min at 70°C. qRT-PCR was performed with gene-specific primers (200 nM) designed for each specie (Table 2) from sequences consensus between the different strains in a 10 μl reaction, using the Light Cycler FastStart DNA MasterPLUS SYBR green (Roche Applied Science, Germany) in a LightCycler® 2.0 System (Roche Applied Science, Germany). All samples were processed for melting curve analysis, amplification efficiency and DNA concentration determination. A mixture of all samples and serial dilutions (10^−1^ to 10^−5^) was used as standard curve. Two different constitutive reference genes were used (*ACT1* and *RDN18-1*) to normalize the amount of mRNA and ensure accuracy, correct interpretation, and repeatability (Starovoytova et al., 2013). The results were normalized by using the normalization factor obtained from geNorm VBA applet (Vandesompele et al., 2002).

## Results

### *Saccharomyces* species differ in tolerance to hyperosmotic and cold stresses

**Figure 1:**
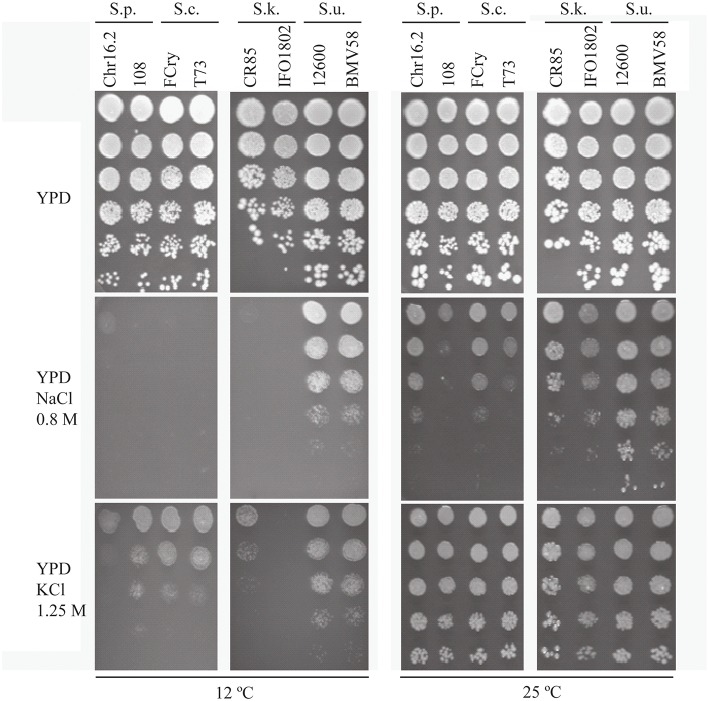
**Osmotolerance of *S. paradoxus* (S.p., Chr16.2, 108), *S. cerevisiae* (S.c. FCry, T73), *S*. *kudriavzevii* (S.k., CR85, IFO1802), and *S. uvarum* (S.u.12600, BMV58) strains at 25 and 12°C**. Serial dilutions were plated in rich media with (YPD NaCl 0.8 M or KCl 1.25 M) or without (YPD) hyperosmotic stress. A representative image of biological triplicates is presented.

The behavior of *S. cerevisiae* and other *Saccharomyces* species interesting for industrial applications was evaluated in response to wine fermentation relevant stresses. We selected hyperosmotic (NaCl 0.8 M and KCl 1.25 M) and a combination of hyperosmotic and cold stresses (12°C), two frequent suboptimal conditions during winemaking. We performed a drop test with two strains of each species (*S. paradoxus, S. cerevisiae, S. kudriavzevii*, and *S. uvarum*) on complete media and compared the growth in the above mentioned conditions (Figure 1). The results revealed that the used stresses have a very different effect on yeast growth depending not only on the species but even on the strain. The stress with KCl 1.25 M is the condition that has less effect on the yeast growth capacity, and the NaCl 0.8 M plus 12°C the most severe stress. The conditions NaCl 0.8 M hyperosmotic stress and KCl 1.25 M at 12°C hyperosmotic-cold stress generated intermediate growth capacity levels. The results showed clearly that the strains can cope better with a higher osmotic stress (KCl 1.25 M) than with the sodium toxicity (NaCl 0.8 M). In hyperosmotic stress mediated by NaCl 0.8 M we observed that *S. uvarum* strains are the ones presenting the highest tolerance to hyperosmotic and a similar observation can be made in the most severe condition (NaCl 0.8 M plus 12°C). The other species showed similar behavior although *S. kudriavzevii* strains showed low growth levels in cold stress condition, especially IFO1802 strain. *S. cerevisiae* and *S paradoxus* strains showed similar growth levels but strain 108 in NaCl 0.8 M at 25°C and strain Chr16.2 in KCl 1.25 M at 12°C presented lower growth levels than *S. cerevisiae* strains.

### Glycerol levels during wine fermentation

**Figure 2:**
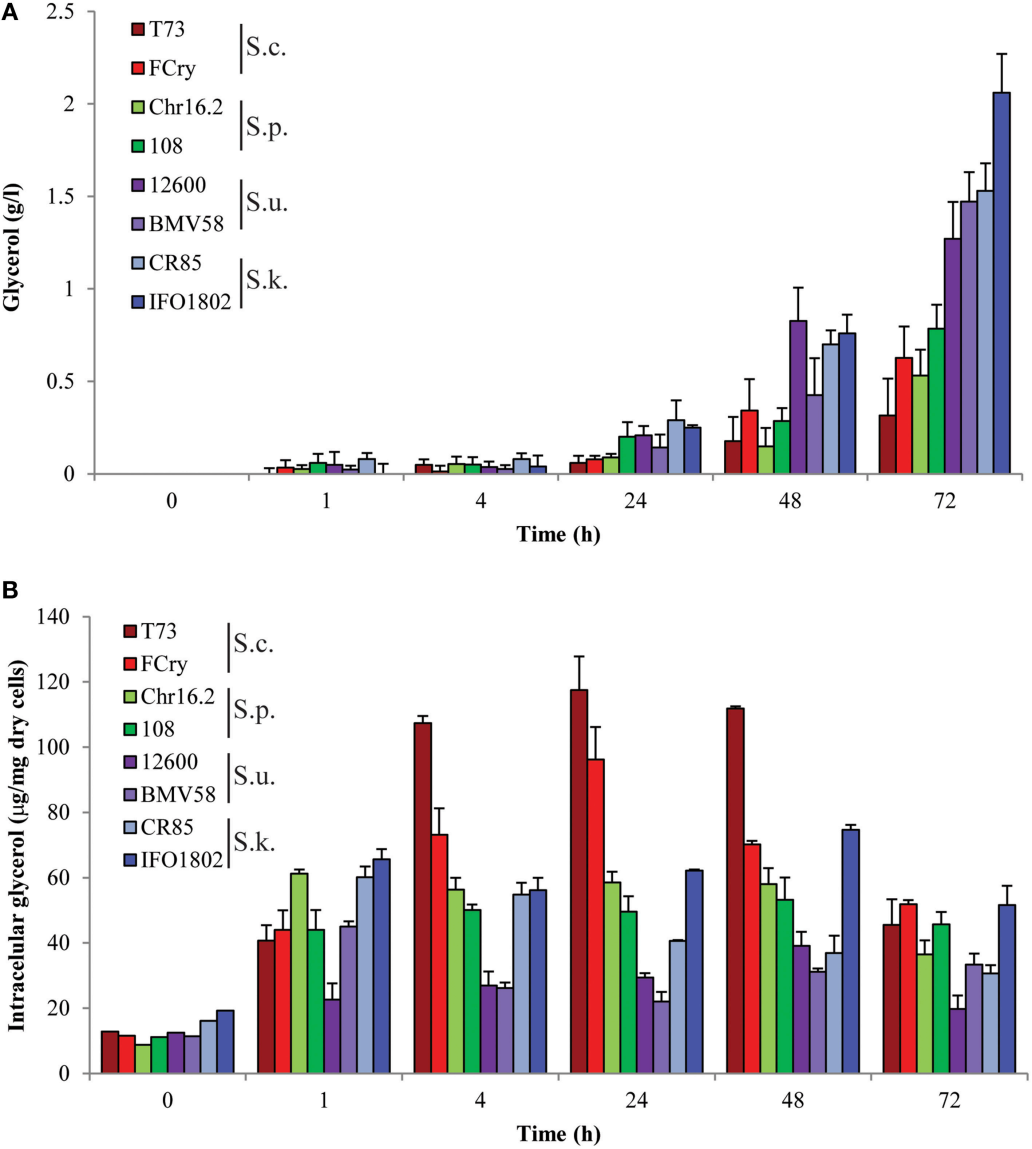
**Microvinification experiments in synthetic must at low temperature with *S. cerevisiae* T73 (dark red) and FCry (light red), *S. paradoxus* Chr16.2 (light green) and 108 (dark green), *S. uvarum* 12600 (dark purple) and BMV58 (light purple), and *S. kudriavzevii* CR85 (light blue) and IFO1802 (dark blue) strains**. Precultured cells were inoculated in synthetic must at 12°C and samples were taken after 0, 1, 4, 24 and 48 h to determine extra **(A)** and intracellular **(B)** glycerol content for each strain. Three independent microvinification bottles were used for each strain and average ± standard deviation is shown.

Since hyperosmotic and also cold stress responses are unequivocally related to glycerol accumulation we wanted to determine glycerol levels during hyperosmotic-cold stress in wine fermentations. Thus we performed wine fermentations in synthetic must with the studied *Saccharomyces* species and strains, and we measured intra- and extracellular amount of glycerol during the first hours and days of the fermentation. In the results presented in Figure 2 we observed two steps regarding glycerol accumulation in *S. cerevisiae* strains. In the first step, glycerol starts to accumulate inside the cell (Figure 2B) immediately after inoculating into the cold-hyperosmotic condition, reaching a maximal value after 24 h. Also, minimal glycerol levels are accumulated in extracellular media in the beginning of our experiment (Figure 2A). In the next 2 days, intracellular glycerol is reduced and tends to recover its original levels whereas extracellular glycerol increases with the time. In the case of *S. paradoxus* and *S. kudriavzevii*, maximal intracellular glycerol accumulation, which are approximately half of those in *S. cerevisiae* strains, occurs in the first hours and levels are maintained during 48 h. Analyzing the intracellular glycerol level (Figure 2B), it is interesting to note that, comparing with the other species, *S. cerevisiae* strains accumulated the higher levels of glycerol between 4 and 48 h of incubation. The *S. uvarum* strains showed the lowest values of intracellular glycerol with a maximal level after 1 h in the case of BMV58 and after 48 h in the case of 12600. Regarding extracellular glycerol (Figure 2A), *S. paradoxus* presented similar levels and accumulation pattern as *S. cerevisiae* and *S. uvarum* and, in addition, *S. kudriavzevii* showed a similar pattern but higher accumulation levels (around five times more). Is interesting to emphasize that *S. uvarum* and *S. kudriavzevii* showed a higher extracellular glycerol accumulation rate compared to the other two species. Interestingly, no extracellular glycerol was observed at time 0 in any species. It should be noted that strains do not show significant growth after 1 or 4 h and maximal yeast biomass was observed at the 24 or 48 h time point except for the IFO1802 that show very low growth level in grape must (Supplementary Figure 1), in concordance with data observed in Figure 1 in osmotic and cold stress conditions.

### Changes in mRNA levels of genes related to glycerol balance during wine fermentation and hyperosmotic stress of different *Saccharomyces* species

**Figure 3:**
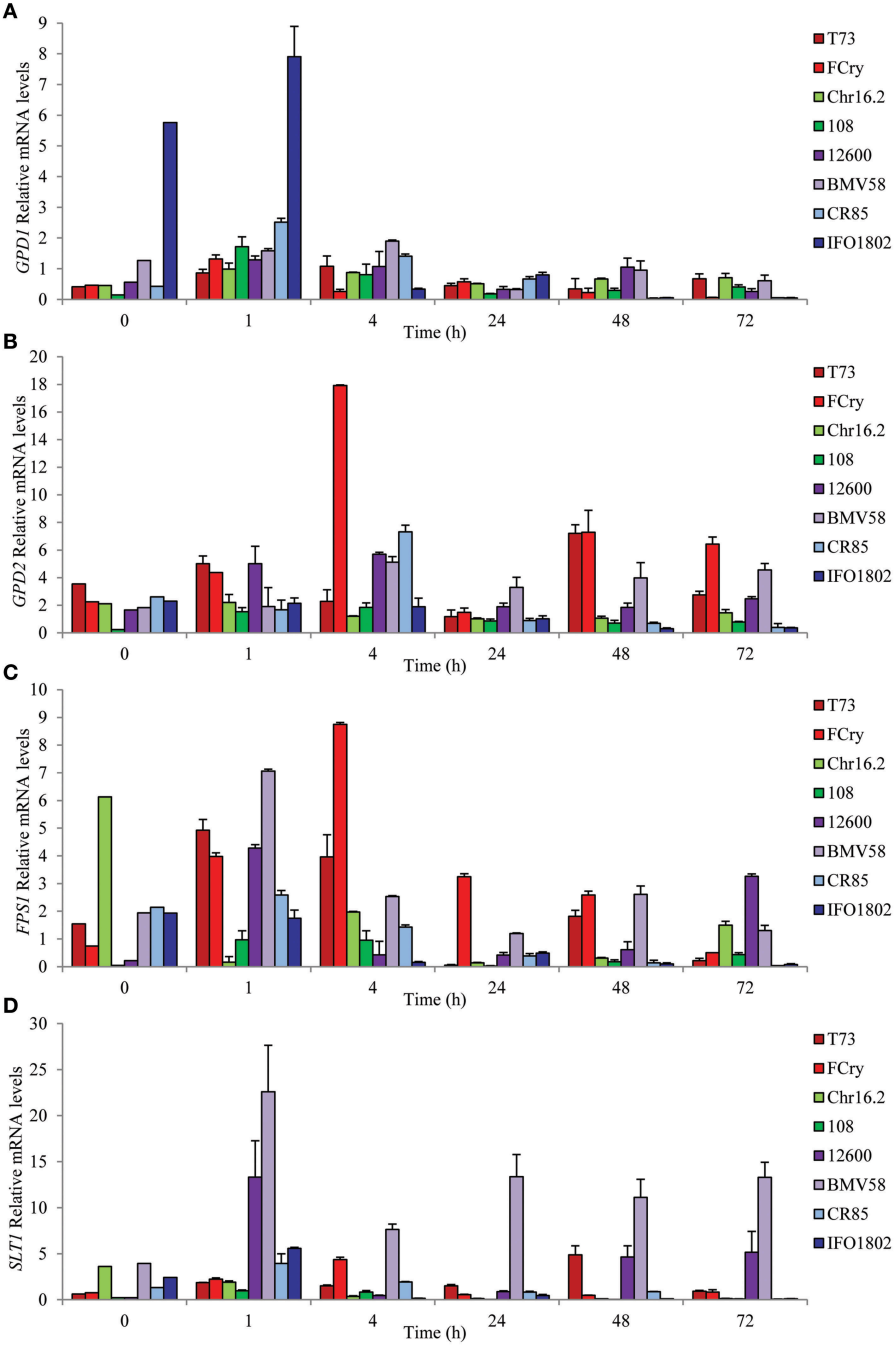
**Expression of glycerol balance related genes during first hours of low temperature microvinifications in synthetic must for *S. cerevisiae* T73 (dark red) and FCry (light red), *S. paradoxus* Chr16.2 (light green) and 108 (dark green), *S. uvarum* 12600 (dark purple) and BMV58 (light purple) and *S. kudriavzevii* CR85 (light blue) and IFO1802 (dark blue) strains**. The genes related to glycerol biosynthesis,*GDP1* **(A)** and *GPD2* **(B)**, and glycerol export, *FPS1* **(C)**, and import, *STL1* **(D)**, were studied. Samples were taken in the first part (0, 1, 4, 24, 48 and 73 h) of synthetic must microvinifications at 12°C. After RNA extraction, expression of the different genes was determined by qPCR and values were normalized with *ACT1* and *RDN18-1* constitutive genes. Three independent microvinification bottles were used for each strain and averages ± standard deviation are shown.

To gain insights on the regulation of glycerol pools balance we studied variation in mRNA levels of key genes related to glycerol biosynthesis (*GPD1* and *GPD2*), efflux (*FPS1*), and influx (*STL1*) in the same wine fermentation conditions described above and in the same strains and species. The results (Figure 3) clearly revealed different patterns and levels of gene expression among the species in all four genes studied. In the case of *GPD1*, all the strains showed a general pattern of induction after the first hour but with marked differences in the expression levels. *S. kudriavzevii* strains showed the highest mRNA levels, specially IFO1802 strain that presented elevated expression of *GPD1* before stress and even more after 1 h of inoculation. For the *GPD2* gene, which is mainly involved in redox balance, some of the strains presented an induction with maximal levels after four (*S. uvarum* strains, FCry, 108, and CR85) or 48 (T73) hours whereas other strains (Chr16.2 and IFO1802) seem to not activate this gene showing low mRNA levels. The *FPS1* gene expression peaked after 1 h (108, CR85, *S. cerevisiae*, and *S. uvarum* strains) or 4 h (Fcry), with the *S. cerevisiae* and *S. uvarum* strains showing the highest levels. The IFO1802, Chr16.2, and *S. kudriavzevii* strains did not showed significant increase of mRNA levels compared to the inoculum. Finally, the *SLT1* gene presented the most variable mRNA levels among the species showing highest values for the *S. uvarum* strains, especially BMV58, with a maximum after 1 h. Other species showed a moderate amount of mRNA with maximum levels after 1 h (*S. kudriavzevii* strains) or 4 h (Fcry). *S. paradoxus* strains showed very low *SLT1* mRNA levels along the experiment. Yeast growth phase does not has a dramatic impact in activation of gene expression in this conditions since the most important inductions were observed at 1 and 4 h, were grow was not observed. The comparison between glycerol content and gene expression results emphasize the importance of *GPD1* and *STL1* in *S. kudriavzevii* and *S. uvarum* respectively, regarding their increased glycerol accumulation (Figure 2A). In the case of *S. cerevisiae* strains, increased *GPD2* levels, especially in Fcry strain, could explain the high intracellular glycerol levels observed in Figure 2B. On the contrary, *FPS1* increased expression does not reflect extracellular glycerol levels in *S. cerevisiae* probably due to the tight regulation of this channel by posttranslational mechanisms.

**Figure 4:**
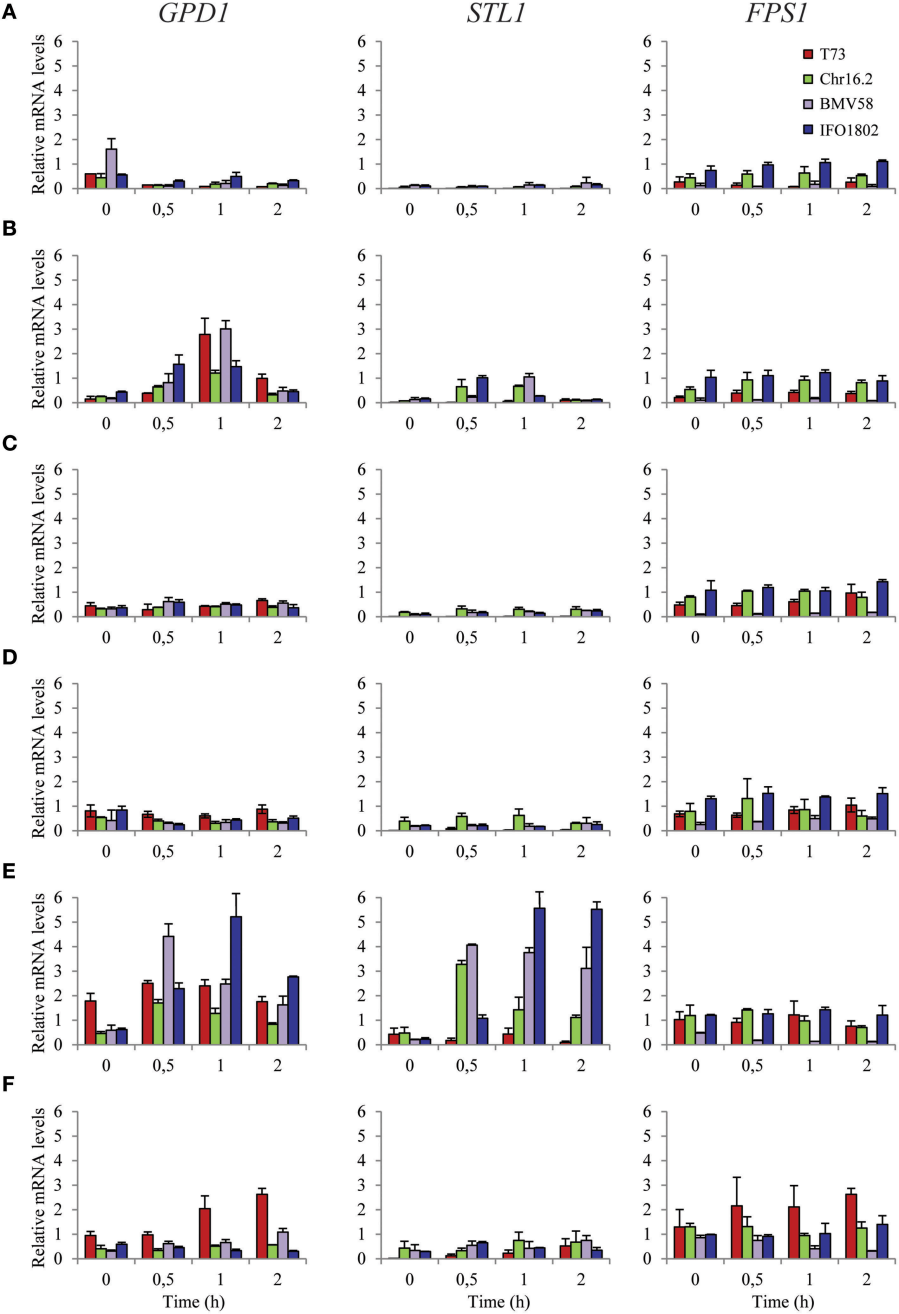
**Expression of glycerol balance related genes of *S. cerevisiae* T73 (red), *S. paradoxus* Chr16.2 (green), *S. uvarum* BMV58 (purple), and *S. kudriavzevii* IFO1802 (blue) strains in various conditions**. The genes related to glycerol biosynthesis (*GDP1*) and glycerol transport (*FPS1* and *STL1*) were studied. Samples from non-stress SC media **(A,D)**, hyperosmotic SC 1 M sorbitol **(B,E)** or hypoosmotic (water) media **(C,F)** cultures were taken after 0, 0.5, 1 and 2 h of the inoculation (from pre grown cultures in SC). The SC media were supplemented with 2% glucose **(A–C)** or 2% mannitol **(D–F)** as a carbon source. After RNA extraction, expression of the different genes was determined by qPCR and values were normalized with *ACT1* and *RDN18-1* constitutive genes. Three independent microvinification bottles were used for each strain and averages ± standard deviation are shown.

To study the regulation of key genes related to intracellular glycerol balance under standard lab conditions (Figure 4) we used a representative strains of each species (T73, Chr16.2, BMV58, and IFO1802) and measured mRNA levels of *GPD1, STL1* and *FPS1* after half, 1 and 2 h of transfer cells to a non-stress SC media (Figure 4A), hyperosmotic SC 1 M sorbitol (Figure 4B) or hypoosmotic (water) media (Figure 4C). In addition, another analog set of experiments were performed but using mannitol as a carbon source (Figures 4D–F), which is a non-fermentable carbon source that complicates the energy supply for cellular processes. No yeast growth was observed during this experiment (results not shown). We can observe that all strains, especially T73 and BMV58, activate *GPD1* 0.5-1 h after hyperosmotic stress (Figure 4B) but is not activated in non-stress conditions (Figure 4A) or hypoosmotic stress (Figure 4C). A similar situation but with higher mRNA levels is observed in presence of mannitol instead of glucose where hyperosmotic stress (Figure 4E) activates *GPD1* gene, especially for BMV58 and IFO1802. In this case, hypoosmotic stress (Figure 4F) does activate the *GPD1* gene in the case of T73 and BMV58. The *STL1* gene reacts with a similar patter as *GPD1* increasing mRNA levels in hyperosmotic stress (Figure 4B) but not upon hypoosmotic stress in the presence of glucose (Figure 4C). *STL1* shows also a similar patter as *GPD1* in presence of mannitol, increasing expression levels after hyperosmotic stress (Figure 4E), though to higher levels compared to glucose (Figures 4B,E). Interestingly, *S. cerevisiae* T73 strain shows very low *STL1* levels in any conditions and no significant activation (Figure 4F). On the contrary, the *FPS1* gene seems to be unresponsive to any condition in all the strains with except for the case of T73 growth in mannitol and hypoosmotic stress (Figure 4F). Similar levels are presented for all strains and conditions although BMV58 presented lower levels that the other strains. Altogether, it is the *STL1* gene whose expression shows the highest level of variation in different conditions and among the species and strains.

### Stl1 functional differences in *Saccharomyces* species

**Figure 5:**
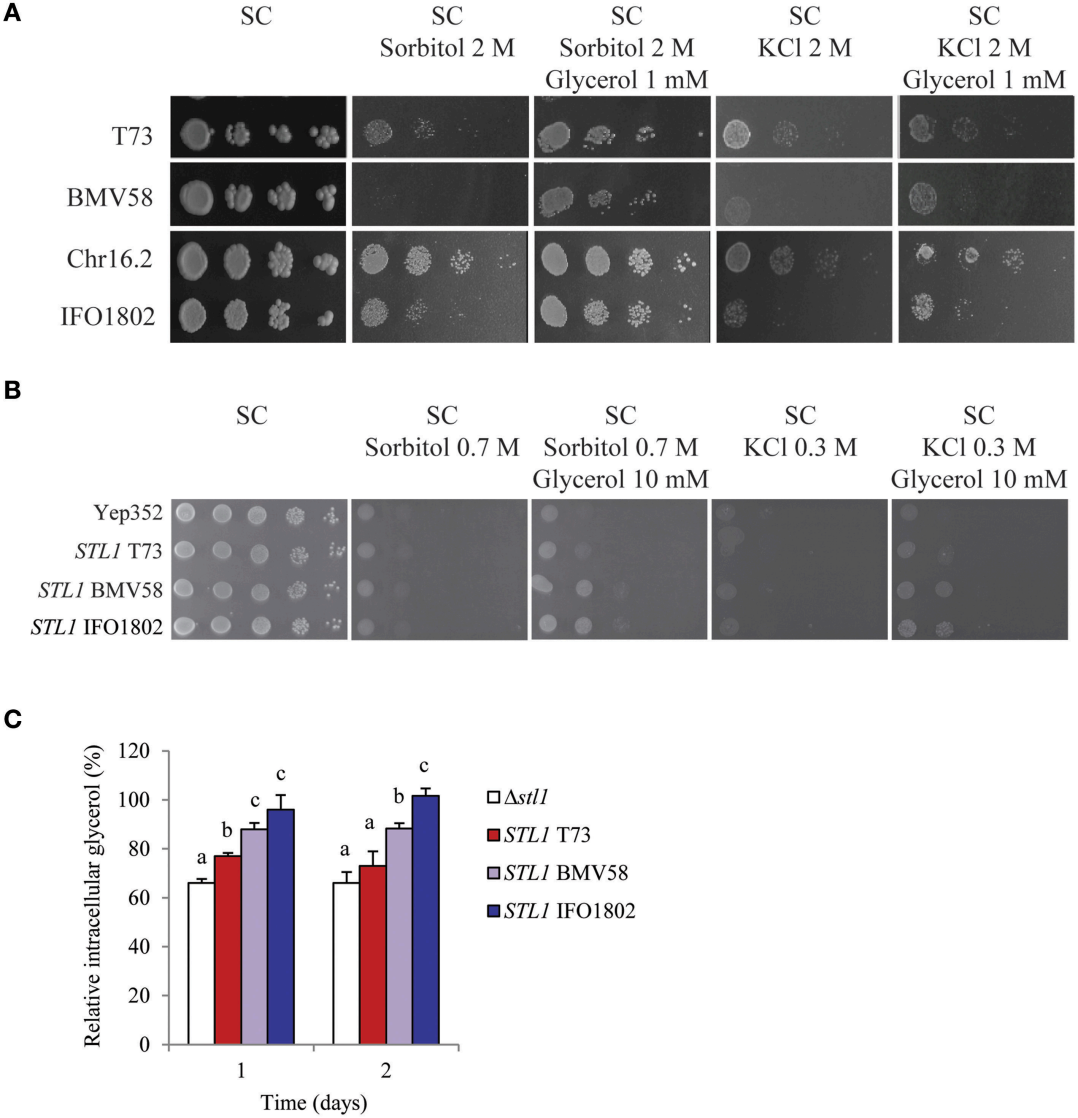
**Importance of glycerol import for osmotolerance of *S. cerevisiae* (T73), *S. uvarum* (BMV58) *S. paradoxus* (Chr16.2), and *S. kudriavzevii* (IFO1802) in drop test assays. (A)** Serial dilutions of the different strains were plated in non-stress media (SC), in hyperosmotic stress media (SC with 2 M sorbitol or 2 M KCl) and in hyperosmotic stress media supplemented with glycerol (1 mM glycerol) **(B)** Growth of *S. cerevisiae* BY4741Δ*stl1*Δ*hog1* strain expressing *STL1* alleles from *S. cerevisiae* (T73), *S. uvarum* (BMV58) or *S. kudriavzevii* (IFO1802) was monitored in drop tests on non-stress media (SC), in hyperosmotic stress media (SC with 0.7 M sorbitol or 0.3 M KCl), and in hyperosmotic stress media supplemented with 10 mM glycerol. A representative image of biological triplicates is presented. **(C)** In the same strains used in **(B)**, intracellular glycerol accumulation was measured collecting samples after 0, 1, or 2 days of growth in SC with 10% glucose. Cells precultured in the same media were inoculated (OD_600_ = 0.3) and incubated at 25°C in 100 ml flasks. Data in time 0 for each strain was considered 100%. Three independent experiments were performed for each strain and averages ± standard deviation are shown. ANOVA with fisher test (*p* < 0.05) was performed and significantly different values are labeled with different letters.

Since *STL1* gene presented important differences in mRNA levels in strains from different *Saccharomyces* species we wanted to study the possible functional differences of this glycerol importer. For that we first compared the growth of a representative strain of *S. cerevisiae* (T73), *S. uvarum* (BMV58), *S. paradoxus* (Chr16.2), and *S. kudriavzevii* (IFO1802) species in conditions where the activity of Stl1 is important (Figure 5A). A drop test with the four strains was performed in non-stress media (SC), in hyperosmotic stress media (SC with 2 M sorbitol or 2 M KCl) and in hyperosmotic stress media supplemented with a very low amount of glycerol (SC with 2 M sorbitol (or 2 M KCl) and 1 mM glycerol). In these conditions, if the cells are able to efficiently import glycerol to the cytosol they have a growth advantage when extracellular glycerol is present, i.e., before they synthesize the necessary amount to counterbalance the external osmotic pressure. The results show that cell growth is affected by hyperosmotic stress conditions proportionally to the osmotic pressure, i.e., more in the presence of 2 M KCl than in the presence of 2 M sorbitol. We can observe that BMV58 is the strain with the lowest and Chr16.2 the highest survival level in both hyperosmotic stress conditions. Interestingly, as shown in the Figure 5A, some strains, as IFO1802 and especially BMV58, benefit from the presence of glycerol in the medium more than others (e.g., T73 and Chr16.2). These results are indicative of different capacity to import glycerol in response to hyperosmotic stress among the studied strains. Is interesting to highlight that osmotolerances in minimal media can be different to complete media (see BMV58 in Figure 1 compared to Figure 5A). This reflects that strains disposition to cope to osmotic stress could be different since complete and minimal media induce very different gene expression programs (Gasch et al., 2000; Miura et al., 2008).

To confirm these Stl1 functional differences we cloned the different *STL1* alleles from T73, BMV58, and IFO1802 strains in an *S. cerevisiae* multicopy plasmid behind a weak and constitutive promoter, and expressed them in a laboratory osmosensitive *S. cerevisiae* strain (BY4741Δ*slt1*Δ*hog1*). As a control, this strain was also transformed with the empty YEp352. Then, the growth of strains was tested in non-stress media (SC), in hyperosmotic-stress media (SC with 0.7 M sorbitol or 0.3 M KCl) and in hyperosmotic-stress media supplemented with extracellular glycerol (SC 0.7 M sorbitol or 0.3 M KCl, and 10 mM glycerol). The results (Figure 5B) showed that the strains with the BMV58 and IFO1802 *SLT1* allele are clearly able to recover growth when they have extracellular glycerol in the presence of a hyperosmotic stress. However, the strain containing the T73 *STL1* allele presented only a minor growth recovery when it can use extracellular glycerol in the presence of a hyperosmotic stress.

We also evaluated the different Slt1 functionality by measuring the intracellular glycerol accumulation of the *S. cerevisiae* strains expressing different *STL1* genes after 1 and 2 days of growth in 10% glucose (Figure 5C) without any additional osmotic agents. The strain with IFO1802 Stl1 was able to recover the original intracellular glycerol levels by importing some of the diffused out glycerol. The strain with BMV58 Stl1 was able to recover more than 80% of the original intracellular glycerol levels. On the contrary, after 2 days the strain with the T73 Stl1 showed intracellular glycerol levels recovery no significantly different than a strain without Slt1. This results points in the same direction of the previous experiments and suggest a low functionality of T73 Stl1 compared with BMV58 and IFO1802.

## Discussion

In this work we studied intracellular glycerol pool balance and regulation in response to stresses that occur upon inoculating wine-related yeast species in grape musts. We have analyzed strains belonging to four species that participate in winemaking directly (*S. cerevisiae, S. uvarum*, and *S. paradoxus*) or through hybrids (*S. kudriavzevii*). A first approach was to compare survival of these species during hyperosmotic and cold-hyperosmotic stress. Other studies have found that *S. cerevisiae, S. uvarum*, and *S. paradoxus* strains have similar tolerance to hyperosmotic stress whereas *S. kudriavzevii* strains show a decreased survival in 15% sorbitol at 30°C (Wimalasena et al., 2014). However, this result is doubtful since *S. kudriavzevii* strains are sensitive to this temperature (Arroyo-López et al., 2010). In our results using 25°C, an optimal temperature for *S. kudriavzevii*, this species shows similar or slightly higher tolerance to some hyperosmotic conditions compared to *S. cerevisiae* and *S. paradoxus*. In contrast, we observed an increased hyperosmotic stress tolerance in *S. uvarum* strains that is even more evident in hyperosmotic-cold stress conditions, where glycerol balance is determinant for cell survival. These results argue in favor to a more efficient handling of intracellular glycerol in *S. uvarum* strains in this condition. On the contrary, hyperosmotic tolerances in *Saccharomyces* species seems to be dependent on the media since *S. uvarum* strain BMV58 shows the lowest hyperosmotic tolerance in minimal media (Figure 5A) instead of the highest tolerance in complete media (Figure 1). All these data point to different strategies in the different species to handle glycerol accumulation in response to hyperosmotic or cold-hyperosmotic stresses.

**Figure 6:**
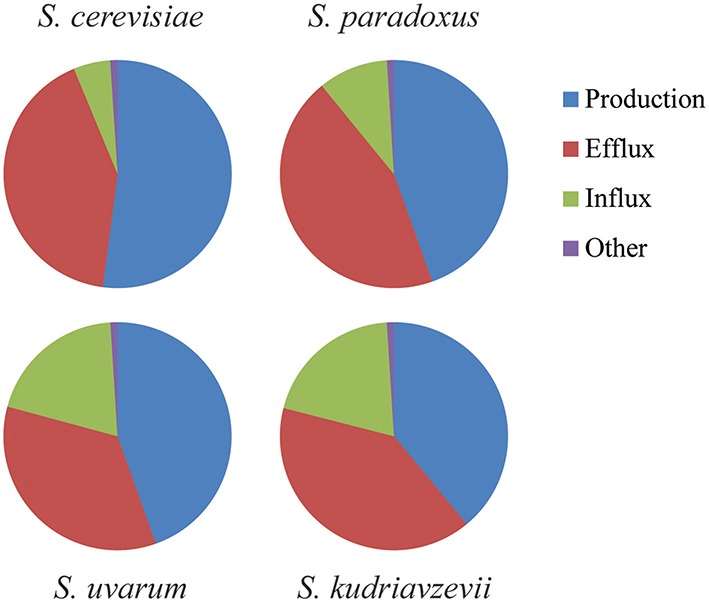
**Schematic representation of the weight of glycerol production, efflux, influx, or other actions regarding glycerol balance after hyperosmotic stress**. This representation compares the dynamics of glycerol accumulation in response to hyperosmotic stress that has been quantitatively analyzed and modeled using physiologic, metabolic, enzymatic, and transcriptomic data of the key actors in *S. cerevisiae* (Petelenz-Kurdziel et al., 2013). Here we compared with the other species (*S. paradoxus, S. uvarum*, and *S. kudriavzevii*) using data provided in this work and others as Oliveira et al. (2014).

In winemaking conditions, cells suffer hyperosmotic or cold hyperosmotic mild stresses that do not affect cell growth capacity in any *Saccharomyces* species (results not shown). This hyperosmotic stress produced by the elevated amount of sugars may determine different lag phase adaptations. In fact, significant differences can be observed in extra and intracellular glycerol levels and also in gene expression of key genes involved in glycerol homeostasis. These data also suggest that the *Saccharomyces* species are using different strategies to face alterations in the osmotic pressure and cold temperatures. In fact this argument is not that surprising since *Saccharomyces* species are genetically quite distant showing coding region identities such as the one showed when comparing human and mouse (85%; Lapidot et al., 2001). The dynamics of glycerol accumulation in hyperosmotic stress has been quantitatively analyzed and modeled using physiologic, metabolic, enzymatic, and transcriptomic data in *S. cerevisiae* (Petelenz-Kurdziel et al., 2013). The strategy of this species consists in a transcriptional activation of *GPD1* to increase glycerol accumulation inside the cell by redirecting glycolytic flux. On the other hand, the glycerol efflux stops by the closing of Fps1channel. These are the principal mechanisms to balance glycerol after a hyperosmotic shock. Glycerol influx and other elements contribute in a minor fraction (Figure 6). From the results of this work and others, we can hypothesize that non-*cerevisiae Saccharomyces* species have changed the weight of the different elements involved in glycerol balance. Based on *STL1* gene activation and Stl1 functionality assays we speculate that *S. uvarum* and *S. kudriavzevii* rely more in the glycerol import to compensate the osmotic pressure when extracellular glycerol is accumulated (Figure 6). This strategy is not exclusive of these species, In fact, it has been shown that the most osmotolerant yeasts species present a very efficient glycerol-import capacity (Lages et al., 1999).

A possible explanation of the different strategies applied by the *Saccharomyces* species to balance glycerol in osmotically non-optimal environments could be amount of intracellular glycerol that cells need to accumulate. We observed that, in our winemaking conditions, *S. cerevisiae* accumulates the highest amount of glycerol in the cells. This promotes the supposition that the other species can partially compensate the osmotic pressure by other means as cell volume changes for example. This will allow them to diversify the mechanisms available to compensate water efflux by using more frequently other elements that can be inefficient in specific situations, for example the glycerol import, which can be useless if there is no glycerol outside the cell. This variation could be consequence of environmental adaptation to different niches. For example, cold stress adaptations could implement glycerol influx to better cope with low temperatures. Future research will shed more light in the effect of other conditions as redox unbalance and anaerobiosis in the glycerol pools in the different species.

In summary, the four species studied show different strategies to survive under osmotic or cold-osmotic stressful conditions (Figure 6). In all species, the balance of intracellular glycerol which depends on the production, efflux, influx and other minor elements is altered in order to increase its levels. However, whereas a species as *S. cerevisiae* relays more in changes in the production levels, others tend to depend more on the variation of the influx as *S. uvarum* or *S. kudriavzevii*.

## Author contributions

RP conceived the study and participated in its design and coordination and draft the manuscript. GMO and JZ performed the experiments and analyzed the results. HS and AQ participated in the design and coordination of the study and in the draft of the manuscript. All authors read and approved the final manuscript.

### Conflict of interest statement

The authors declare that the research was conducted in the absence of any commercial or financial relationships that could be construed as a potential conflict of interest.

## Supplementary material

Supplementary Figure 1 **Yeast biomass accumulation during low temperature microvinifications in synthetic must for *S. cerevisiae* T73 (dark red) and FCry (light red), *S. paradoxus* Chr16.2 (light green) and 108 (dark green), *S. uvarum* 12600 (dark purple) and BMV58 (light purple), and *S. kudriavzevii* CR85 (light blue) and IFO1802 (dark blue) strains**.

## Abbreviations

*GPD1*: glycerol-3-phosphate dehydrogenase 1 gene
*GPD2*: glycerol-3-phosphate dehydrogenase 2 gene
*STL1*: glycerol proton symporter gene
*FPS1*: glycerol channel gene
*ACT1*: actin gene
*RDN18-1*: 18S ribosomal RNA gene
qRT-PCR: quantitative real-time PCR
dithiothreitol: DTT.
