# Supplementary figures and images for: Alternative Glycerol Balance Strategies among Saccharomyces Species in Response to Winemaking Stress
Source: Front Microbiol. 2016 Mar 31;7:435. doi: 10.3389/fmicb.2016.00435 (PMC4814467; doi:10.3389/fmicb.2016.00435)

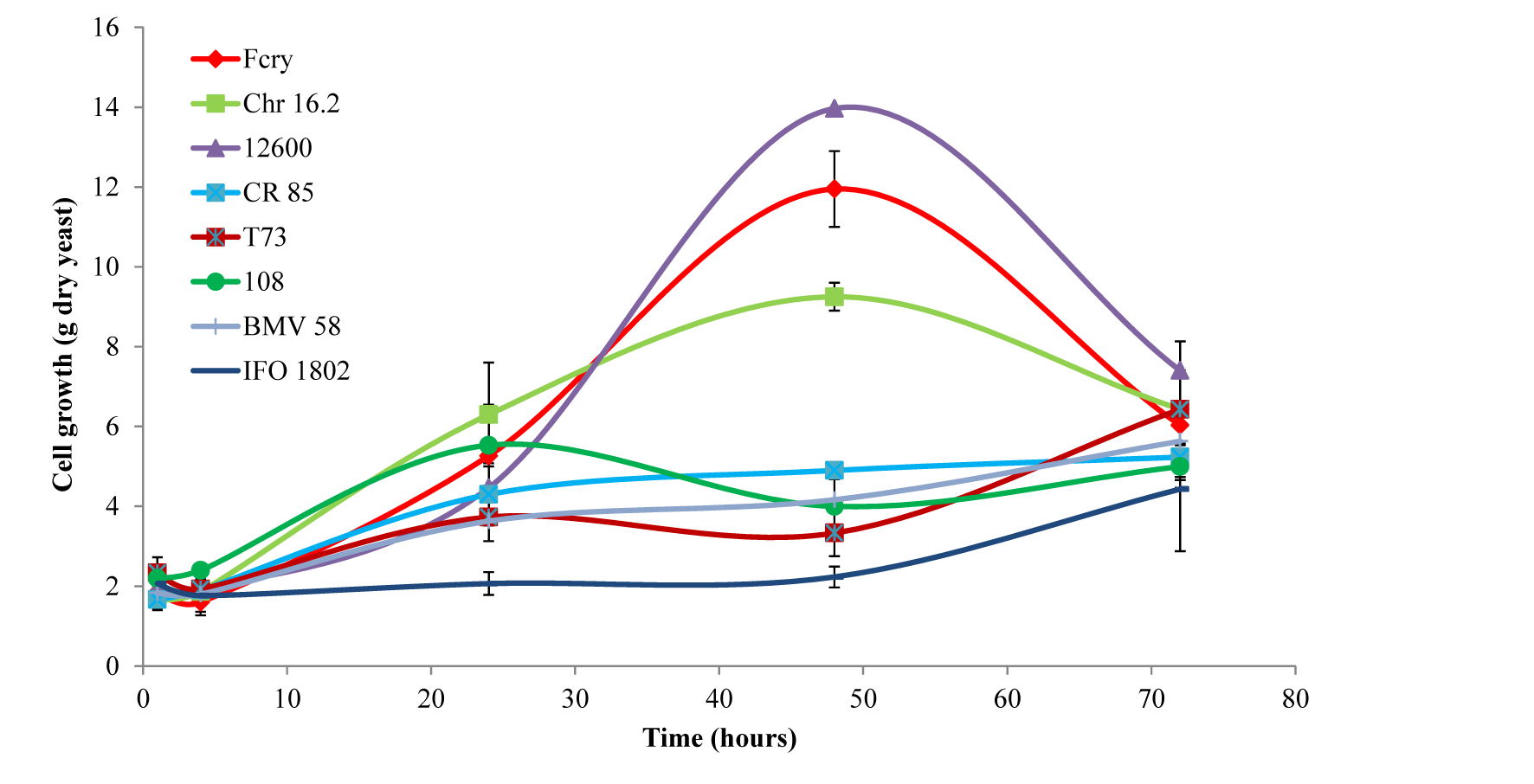

Supplement: Supplementary Figure 1 — Yeast biomass accumulation during low temperature microvinifications in synthetic must for S. cerevisiae T73 (dark red) and FCry (light red), S. paradoxus Chr16.2 (light green) and 108 (dark green), S. uvarum 12600 (dark purple) and BMV58 (light purple), and S. kudriavzevii CR85 (light blue) and IFO1802 (dark blue) strains. [file Image1.TIF]
